# Evaluation of retrieval accuracy and visual similarity in content-based image retrieval of chest CT for obstructive lung disease

**DOI:** 10.1038/s41598-024-54954-5

**Published:** 2024-02-26

**Authors:** Jooae Choe, Hye Young Choi, Sang Min Lee, Sang Young Oh, Hye Jeon Hwang, Namkug Kim, Jihye Yun, Jae Seung Lee, Yeon-Mok Oh, Donghoon Yu, Byeongsoo Kim, Joon Beom Seo

**Affiliations:** 1 Department of Radiology and Research Institute of Radiology, Asan Medical Center, University of Ulsan College of Medicine, 86 Asanbyeongwon-Gil, Songpa-Gu, 05505 Seoul, Korea; 2 Department of Radiology, Kyung Hee University Hospital at Gangdong, College of Medicine Kyung, Hee University, Seoul, Korea; 3 Department of Convergence Medicine, Biomedical Engineering Research Center, Asan Medical Center, University of Ulsan College of Medicine, Seoul, Korea; 4 Department of Pulmonary and Critical Care Medicine, Asan Medical Center, University of Ulsan College of Medicine, Seoul, Korea; 5 Coreline Soft, Co., Ltd., Seoul, Korea

**Keywords:** Chronic obstructive pulmonary disease, Computed tomography, Machine learning, Content-based image retrieval, Respiratory tract diseases, Computed tomography, Machine learning

## Abstract

The aim of our study was to assess the performance of content-based image retrieval (CBIR) for similar chest computed tomography (CT) in obstructive lung disease. This retrospective study included patients with obstructive lung disease who underwent volumetric chest CT scans. The CBIR database included 600 chest CT scans from 541 patients. To assess the system performance, follow-up chest CT scans of 50 patients were evaluated as query cases, which showed the stability of the CT findings between baseline and follow-up chest CT, as confirmed by thoracic radiologists. The CBIR system retrieved the top five similar CT scans for each query case from the database by quantifying and comparing emphysema extent and size, airway wall thickness, and peripheral pulmonary vasculatures in descending order from the database. The rates of retrieval of the same pairs of query CT scans in the top 1–5 retrievals were assessed. Two expert chest radiologists evaluated the visual similarities between the query and retrieved CT scans using a five-point scale grading system. The rates of retrieving the same pairs of query CTs were 60.0% (30/50) and 68.0% (34/50) for top-three and top-five retrievals. Radiologists rated 64.8% (95% confidence interval 58.8–70.4) of the retrieved CT scans with a visual similarity score of four or five and at least one case scored five points in 74% (74/100) of all query cases. The proposed CBIR system for obstructive lung disease integrating quantitative CT measures demonstrated potential for retrieving chest CT scans with similar imaging phenotypes. Further refinement and validation in this field would be valuable.

## Introduction

Due to substantial heterogeneity in physiological and imaging characteristics, therapy response, and disease course, including disease progression and mortality in patients with chronic obstructive pulmonary disease (COPD), there have been continuous efforts to characterize and define subtypes of COPD with distinct patterns of emphysema and airway disease in the past decade^1^. Not all, but there are subgroups in which such efforts may afford a marked clinical benefit^2–4^. Chest computed tomography (CT) imaging has revolutionized the diagnostic approach for COPD by defining phenotypes. Recognizing these different phenotypes in COPD and reclassifying COPD patient severity based on imaging measurements of the pathologies responsible for symptoms and progression, may serve as an initial move towards to start personalized or, at least, optimized therapies^5^. Chest CT allow emphysema classification and quantification, as well as quantification of other abnormalities, including bronchial wall thickening, air trapping reflecting small airway disease, bronchiectasis, and pulmonary vessels^6–8^. Quantitative CT measures in patients with COPD have demonstrated their relationship to lung function parameters, clinical symptoms, exacerbation rates, and mortality^9–15^. The commonly recognized clinical phenotypes include chronic bronchitis, emphysema, COPD-asthma, and frequent exacerbators. Patients in each phenotype group share clinical characteristics and, importantly, have similar responses to existing treatments^16^. For instance, patients with chronic bronchitis are good responders and the only candidates for phosphodiesterase-4 inhibitors, while those with overlapping COPD-asthma phenotype show better responses to inhaled corticosteroids^17^. Therefore, in the initial evaluation of patients with COPD, the objective identification of those with similar clinical characteristics and determination of their clinical outcomes might inform clinical decision-making.

Content-based image retrieval (CBIR) is an image search engine with tools for classifying, indexing, and retrieving images with similar appearances from a database. CBIR matches the visual contents of the query image, an input, with those in the archive; the closeness in visual similarity in terms of image feature vectors provides a basis for identifying images with similar appearances^18,19^. To apply CBIR in obstructive lung diseases, the automated quantitative measures for emphysema, airway, and vessels extracted from chest CT can be integrated into the classification and measurement of the similarity index of the CBIR system to find similar patients who share similar phenotype of disease, which might aid in diagnostic evaluation and decision-making in patients with COPD. In this study, we developed a fully automated CBIR system for obstructive lung diseases by incorporating quantitative measures. We assessed the performance of this system for retrieving similar chest CT images as query CT images in patients with obstructive lung diseases and visually assessed the similarities between the retrieved and query CT scans.

## Materials and methods

### Study cohorts

All subjects were selected from the Korean Obstructive Lung Disease (KOLD) cohort studies, which were prospective longitudinal studies of patients with obstructive lung disease from the pulmonary clinics of 17 centers in South Korea. The KOLD cohort study included patients from May 2005 to October 2013. The details of the KOLD cohort study have been published previously^20^ and also demonstrated in Supplementary material. Briefly, the study cohort enrolled patients aged > 18 years with chronic respiratory symptoms as well as one or both of airflow limitation or bronchial hyper-responsiveness.

**Figure 1:**
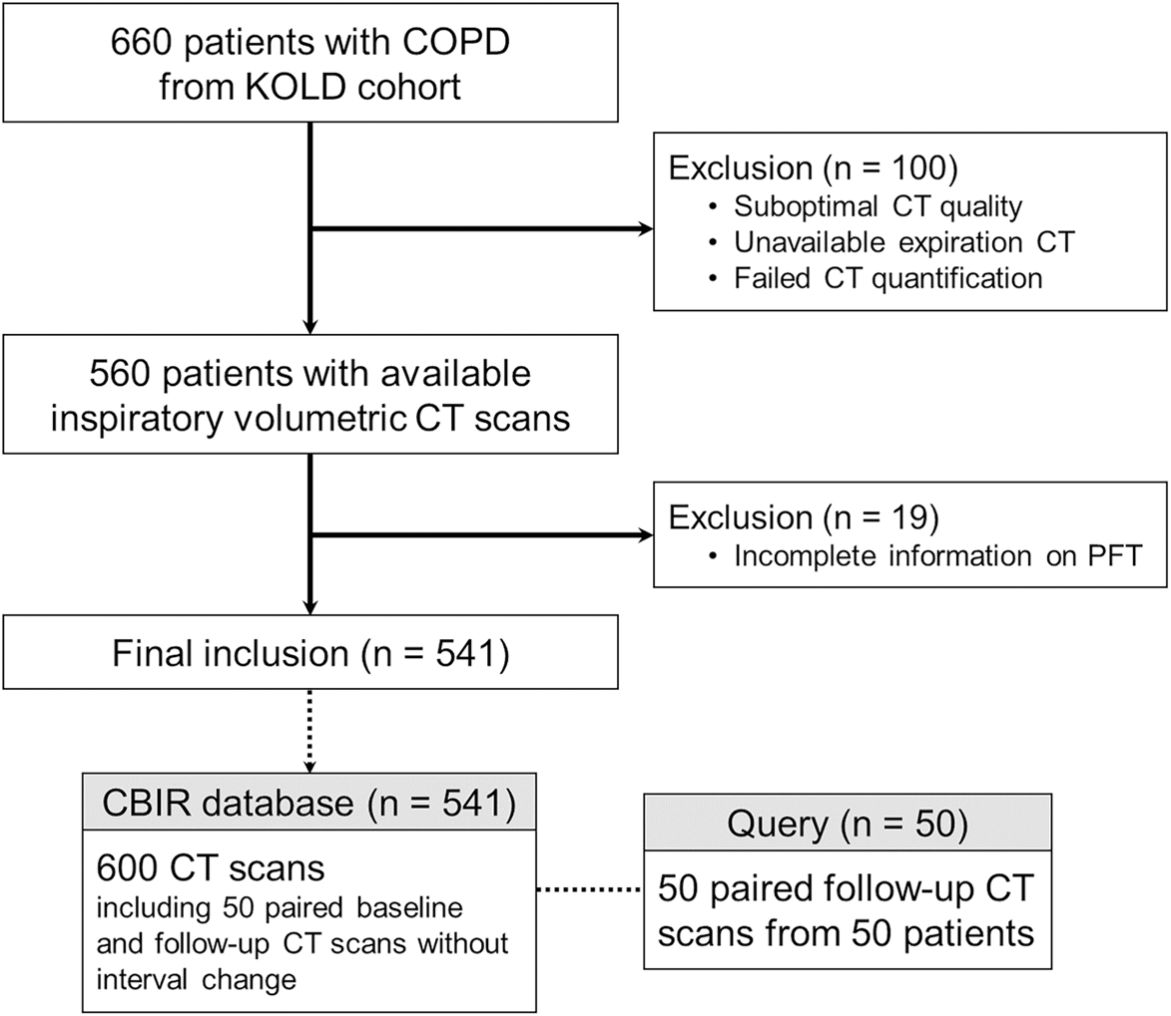
Study flow diagram. COPD—chronic obstructive pulmonary disease; KOLD—Korean Obstructive Lung Disease; CT—computed tomography; PFT—pulmonary function test; CBIR—content-based image retrieval.

After enrollment in the KOLD cohort, 541 patients who underwent volumetric chest CT (3D CT scans with sub-millimeter near isotropic resolution) with full inspiration and pulmonary function test (PFT) were included in the present study and formed the database for the CBIR system. The database contained a total of 600 volumetric CT scans. Among these patients, follow-up CT scans of 50 patients who had initial and follow-up chest CT scans and without significant interval changes in findings between those two CT scans on visual assessment were classified as the query dataset (Fig. 1). The stability of the CT findings between initial and follow-up CT scans was reviewed and confirmed by a thoracic radiologist (S.M.L. with 16 years of experience in thoracic imaging). For the remaining 500 scans, 18 CT scans (two distinct scans per patient) came from nine patients, with each scan taken at a different time point which was not identical (not included as query dataset) and 482 CT scans from 482 patients (one scan per patient). The present study was approved by the Institutional Review Board (No. 2017-1067) of Asan Medical Center and by the Institutional Review Boards of the other 16 participating hospitals. Written informed consent was obtained from all patients. All methods were performed in accordance with the relevant guidelines and regulations.

### CT acquisition and quantitative analysis

We performed volumetric chest CT scans at full inspiration for all patients on 64- or 16-multidetector CT scanners (Somatom Sensation 16 or Definition AS, Siemens Healthineers, Erlangen, Germany; Philips Brilliance 40 or 64, Philips Medical System, Best, the Netherlands). The scan parameters were as follows: tube voltage, 140 kV; tube current, 100–135 effective mA without dose modulation; slice thickness, 0.6–0.75 mm; and reconstruction intervals, 0.5 mm. All images were analyzed using fully automated segmentation software (Aview, Coreline Soft, Seoul, Korea). Fully-automated quantification was performed for the following five feature categories: emphysema volume, emphysema size, airway wall thickness, and numbers of peripheral pulmonary vessels. The quantification of emphysema on CT was based on lung densitometry by determining the relative area of the lungs below -950 Hounsfield units (HU) on inspiration CT (ie, %LAA-950 or emphysema index [EI])^21^. The size variation of emphysema was assessed using the D-slope value by applying a three-dimensional size-based emphysema clustering technique^22^. For the calculation of D-slope, the diameter of the emphysema cluster was plotted against the cumulative number of lesions (number of each lesion diameter) on a log–log scale. The slope (D-slope) of these linear relationships was calculated, with a steeper slope (increase in absolute D value) indicating a smaller emphysema size.

A standardized measure for airway wall thickness was analyzed for each patient by obtaining the Pi10, the square root of the wall area of the theoretical airway with an internal perimeter of 10 mm^23^. We obtained the Pi10 value by plotting the obtainable values of the internal perimeter and the square root of the wall area of the 3rd (lobar level)–8th branches of the bronchi in the whole lungs. The software automatically detected the airway lumens, magnified the images tenfold, and detected the inner and outer boundaries of airway walls using the integral-based half-band method. The details of the airway measurement algorithms for the integral-based half-band method have been described previously^24^. The pulmonary vascular morphology was assessed by measuring the total numbers of aggregate blood vessels < 5 mm^2^ in cross-section (VN_<5 mm_) in the lung surface area 12 mm distant from the pleural surface^25^. EI and VN_<5 mm_ were analyzed for whole lung, right and left lung and each five lobe. Pi10 and D-slope were analyzed for whole lung, right and left lung.

### Development of the CBIR for obstructive lung diseases

**Figure 2:**
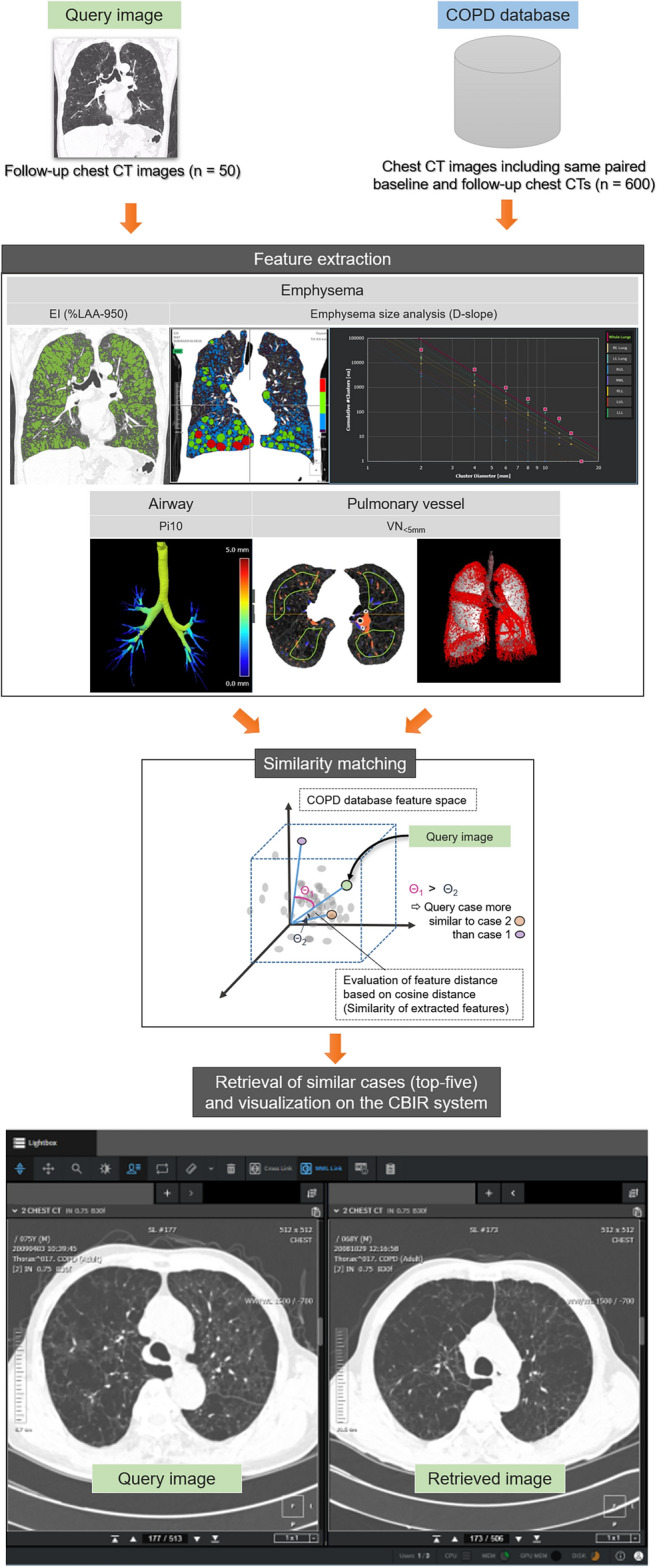
Schematic overview of the content-based image retrieval (CBIR) system for obstructive lung diseases. CT—computed tomography; EI—emphysema index; %LAA-950—percentage of low attenuation areas less than a threshold of -950 Hounsfield units; Pi10—square root of the wall area of a hypothetical airway with an internal perimeter of 10 mm; VN_<5 mm_—the total numbers of aggregate blood vessels with < 5 mm^2^ in cross-section in the lung surface area 12 mm distant from the pleural surface.

**Figure 3:**
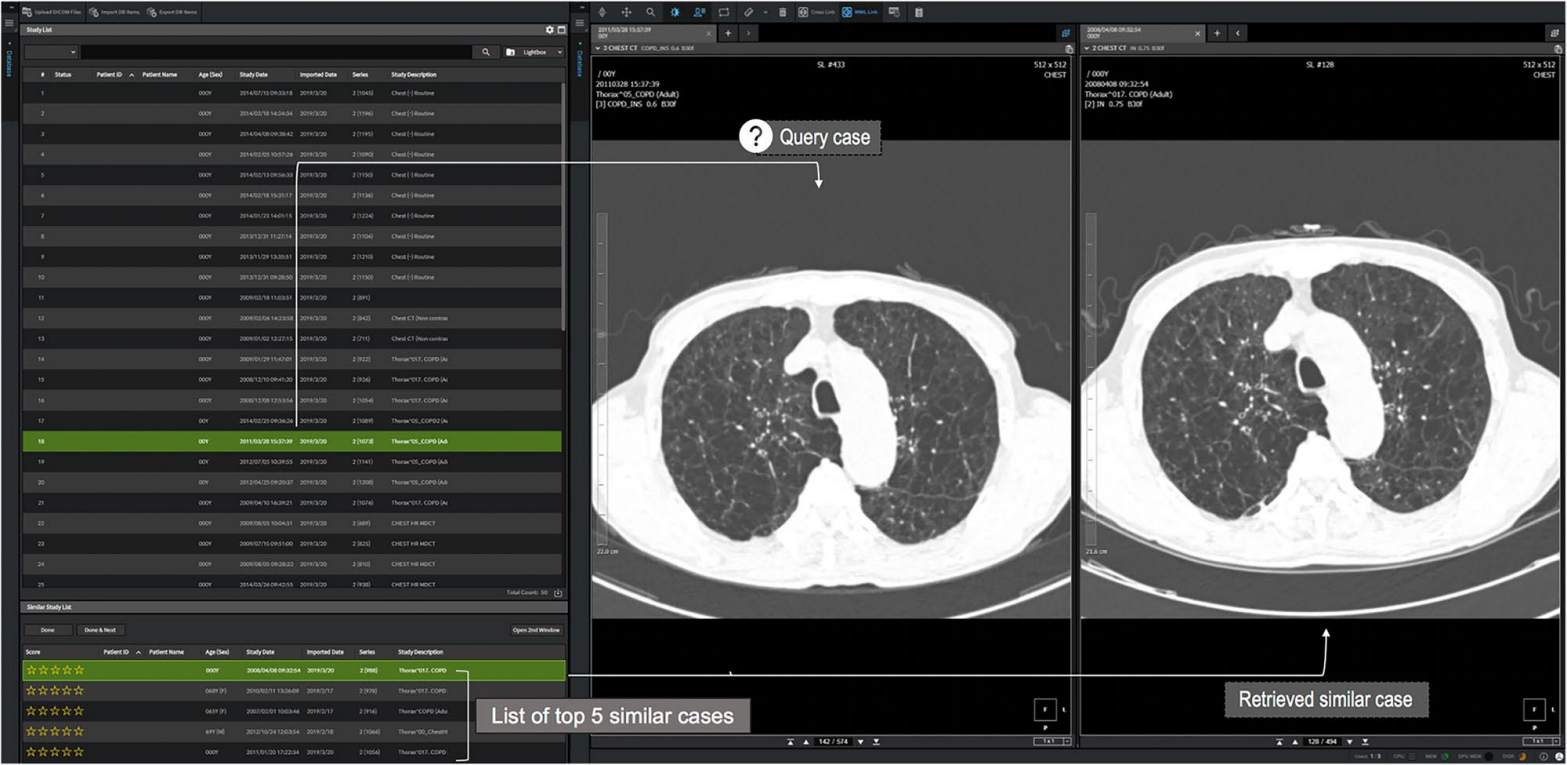
User interface of the CBIR system. CBIR, content-based image retrieval.

Feature extraction was performed in all CT scans in the database through the aforementioned process. The results were indexed in the CBIR system. We normalized each quantitative feature by dividing it by the 95th percentile. The similarities between chest CT scans were compared by measuring the cosine distances among the four feature vectors; the most similar images had the least distance between their feature vectors and vice versa. Finally, the CBIR system retrieved the top five similar images from the database for the given query CT scan in descending order according to the calculated similarities to the query images and displayed the retrievals in the dedicated in-house user interface (Figs. 2 and 3). In addition to our primary features, we also explored the potential of incorporating the air trapping index (ATI) to assess small airway disease by comparing densities using co-registration of the ATI; however, as this did not improve the retrieval performance of similar cases, and adding this variable would render the CBIR system inapplicable to patients without expiratory CT scans, ATI was not included in the finally proposed CBIR system in this study. The details and results of our experimentation with the ATI are elaborated in the Supplementary material.

### Assessment of the retrieval accuracy of the CBIR system

For assessment of the accuracy of the CBIR in retrieving similar CT images, we defined similar CT images as the pairs of chest CT images without change in CT findings showing the stability of the parenchymal abnormalities. The query cases consisted of 50 follow-up CT scans from 50 patients, scanned approximately 3 years after the initial CT scans, within a range of 36–38 months from the initial CT scans whose stability was confirmed by an expert thoracic radiologist. Therefore, the query cases in the database included the CT scans of baseline pairs. For a given query CT scan, the top five similar CT scans were retrieved from the database according to the calculated similarities. To assess the retrieval accuracy of the CBIR, we assessed the rates of retrieving the same pairs of query CT scans among the top 1–5 retrieved CT scans. To evaluate the effect of each feature on retrieval accuracy, the median values of each feature in the 50 query cases were evaluated as a threshold to divide the cases into the top 50% and bottom 50% values.

### Reader assessments of the visual similarity of the retrieved images

Two experienced thoracic radiologists (H.J.H., and S.Y.O. with 15, and 12 years of experience, respectively) independently assessed the visual similarities between the five retrieved CT scans and the query CT. The query and retrieved CT scans were displayed and compared individually and side-by-side using the dedicated in-house interface (Coreline Soft, Seoul, Korea). The five retrieved CT scans were displayed in random order next to the query CT (not in the order of the calculated similarity). The readers were blinded to the patients’ clinical information including age, smoking history, and PFT results. The similarities were subjectively graded using a five-point scoring system according to the emphysema amount, size, and distribution including craniocaudal distribution and the predominant pattern among centrilobular, panlobular and paraseptal emphysema, as well as the degree of bronchial wall thickening. The similarity scores were defined as follows: score 5 (all features are similar), score 4 (two or three similar features), score 3 (one similar feature), score 2 (no features are similar) and score 1 (difficult to evaluate due to little emphysema and bronchial wall thickening). The radiologist who initially evaluated the CT scans for study inclusion did not participate in the visual assessment of feature similarities.

### Statistical analysis

Data are expressed as numeric values with percentages, means with standard deviation, or medians with interquartile range (IQR). The retrieval accuracy was evaluated by grouping the patients according to their median values for each feature and comparing them between different groups using Fisher's exact tests. For the similarity scores of two readers, the mean and confidence interval of similarity scores for each reader and pooled data of two readers were calculated using generalized estimating equation with logit and identified link function. Interreader agreements of similarity scores of two readers were evaluated using the weighted k statistics and intraclass correlation coefficient (ICC). The statistical analyses were performed using IBM SPSS Statistics for Windows, version 23.0 (IBM Corp.) and Stata software, version 16.0 (Stata). For all tests, *P* < 0.05 indicated statistical significance.

## Results

### Patient characteristics

The CBIR database included 541 patients (mean age 66.9 ± 8.5 years; 500 men and 41 women) (Table 1). The mean age of the 50 query cases was 67.9 ± 7.0 years (45 men and 5 women), and the median interval between the initial and follow-up CT examinations was 36 months (range, 34.6–38.0 months).

**Table 1:** Clinical characteristics of the study patients.

Characteristics	All patients (n = 541)	Query (n = 50)
Sex		
Male	500 (92.4)	45 (90.0)
Female	41 (7.6)	5 (10.0)
Age, years*	66.9 ± 8.5	67.9 ± 7.0
Smoking, pack-years^†^	41 (25–54)	42 (31–54)
Pulmonary function test		
FEV1*	53.7 ± 16.9	53.5 ± 15.8
FVC*	78.1 ± 13.6	80.0 ± 16.4
FEV1/FVC*	48.3 ± 12.7	48.6 ± 10.5
FEF_25−75_*	22.6 ± 13.0	23.7 ± 12.5
TLC*	96.9 ± 18.2	100.4 ± 12.6
RV*	104.1 ± 51.8	115.4 ± 36.0
DL_CO_*	77.4 ± 23.0	86.7 ± 27.2
6MWD, m*	421 ± 89	438 ± 76

Unless otherwise indicated, the data are the numbers of patients, with the percentages shown in parentheses.

*Data are mean ± standard deviation.

^†^Data are median, and interquartile ranges are given in parentheses.

DL_CO_, diffusing capacity of the lung for carbon monoxide; FEF_25–75_, forced expiratory flow between 25 and 75% of FVC; FEV1, expiratory volume in 1 s; FVC, forced vital capacity; RV, residual volume; TLC, total lung capacity; 6MWD, 6-min walk distance.

Among quantitative CT parameters, the mean values for each index were 12.2 ± 13.1 for EI, 5.1 ± 1.5 for D-slope, 4.0 ± 0.8 for Pi10, and 0.6 ± 0.1 for peripheral vessel volume in all patients (Table 2). The baseline and follow-up paired CT scans, which consisted of the database and query cases, respectively, showed no significant differences in CT indices between baseline and follow-up CT scans for all five quantitative parameters (all *P* > 0.05; Table 2). The mean values of each index in the query CTs were 11.8 ± 12.2 for EI, 4.9 ± 1.1 for D-slope, 4.3 ± 0.9 for Pi10, and 0.6 ± 0.1 for peripheral vessel volume.

**Table 2:** Clinical characteristics and CT parameters of study patients.

Characteristics	All patients (n = 550)	Paired CT		*P* value*
		Database Baseline CT (n = 50)	Query Follow-up CT (n = 50)	
EI	12.2 ± 13.1	11.2 ± 12.1	11.8 ± 12.2	.76
D-slope	5.1 ± 1.5	5.0 ± 1.1	4.9 ± 1.1	.72
Pi10	4.0 ± 0.8	4.6 ± 1.0	4.3 ± 0.9	.14
Peripheral vessel volume*	0.6 ± 0.1	0.6 ± 0.1	0.6 ± 0.1	.85

Data are mean ± standard deviation.

*Comparisons between the CT indices of the baseline and follow-up CT scans.

CT, computed tomography; ATI, air-trapping index; EI, emphysema index; Pi10, square root of the wall area of a hypothetical airway with an internal perimeter of 10 mm.

### Retrieval accuracy of the CBIR system

**Figure 4:**
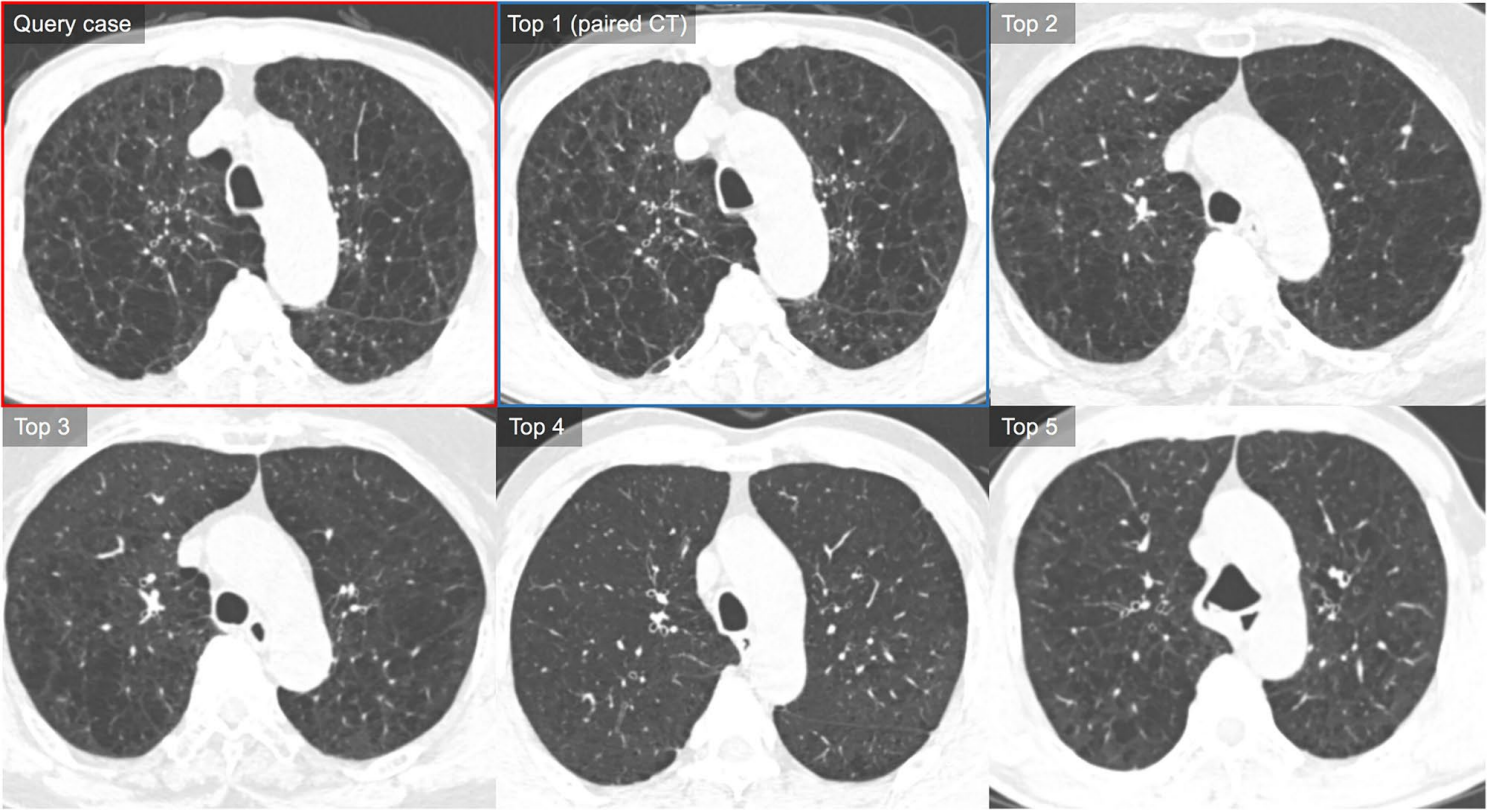
Representative case of the use of the content-based image retrieval (CBIR) system for chronic obstructive pulmonary disease. The query CT shows confluent centrilobular emphysema in both lungs with mild bronchial wall thickening (LAA = 23.1, D-slope = 4.3, PVV = 0.70, and Pi10 = 4.0). CBIR system retrieves five similar CTs based on the measured similarity in descending order. The query case (follow-up CT) is outlined with a red box and its paired case (a pair of baseline CT without interval change compared with the query case) among the retrievals is outlined with a blue box. Our CBIR system retrieved the baseline pair of query CTs from the database as the top 1 retrieval. CT images of top 2 to top 5 retrievals also show quite similar CT features showing mainly confluent or moderate centrilobular emphysema. In the visual similarity assessment, two chest radiologists rated the similarity scores of the top 1–5 as 5, 4, 4, 4, and 3, respectively, and the other chest radiologist as 5, 4, 5, 4, and 3.

The rates of retrieving the same pairs of query CTs in the top 1–3 and top 1–5 images were 60.0% (30/50) and 68.0% (34/50), respectively (Table 3 and Fig. 4). The thresholds of each feature were 7.4% for EI, 4.7 for D-slope, 4.3 for Pi10, and 0.62 for VN_<5 mm_. Regarding the severity of emphysema, the rate of retrieving the same pairs of query CT scans in the top 3 retrieval was slightly higher in patients with EI > 7.4% compared to those with EI ≤ 7.4% (retrieval rates, 72.0% [18/25] vs. 48.0% [12/25]; *P* = 0.09; Fig. 4). No significant difference was observed in retrieval accuracies for D-slope, Pi10, and VN_<5 mm_ (all *P* > 0.05).

**Table 3:** Retrieval accuracy of the content-based image retrieval (CBIR) system.

Rate of retrieval	Total (n = 50)
Presence of the same pair in retrieved cases (%)	
Top 1	20 (40.0, 95%CI 24.4–61.8)
Top 1–3	30 (60.0, 95%CI 40.5–85.7)
Top 1–5	33 (66.0, 95%CI 45.4–92.7)

Data are the numbers of patients, with percentages in parentheses.

CI, confidence interval.

### Reader assessments of the visual similarity of the image retrievals

**Figure 5:**
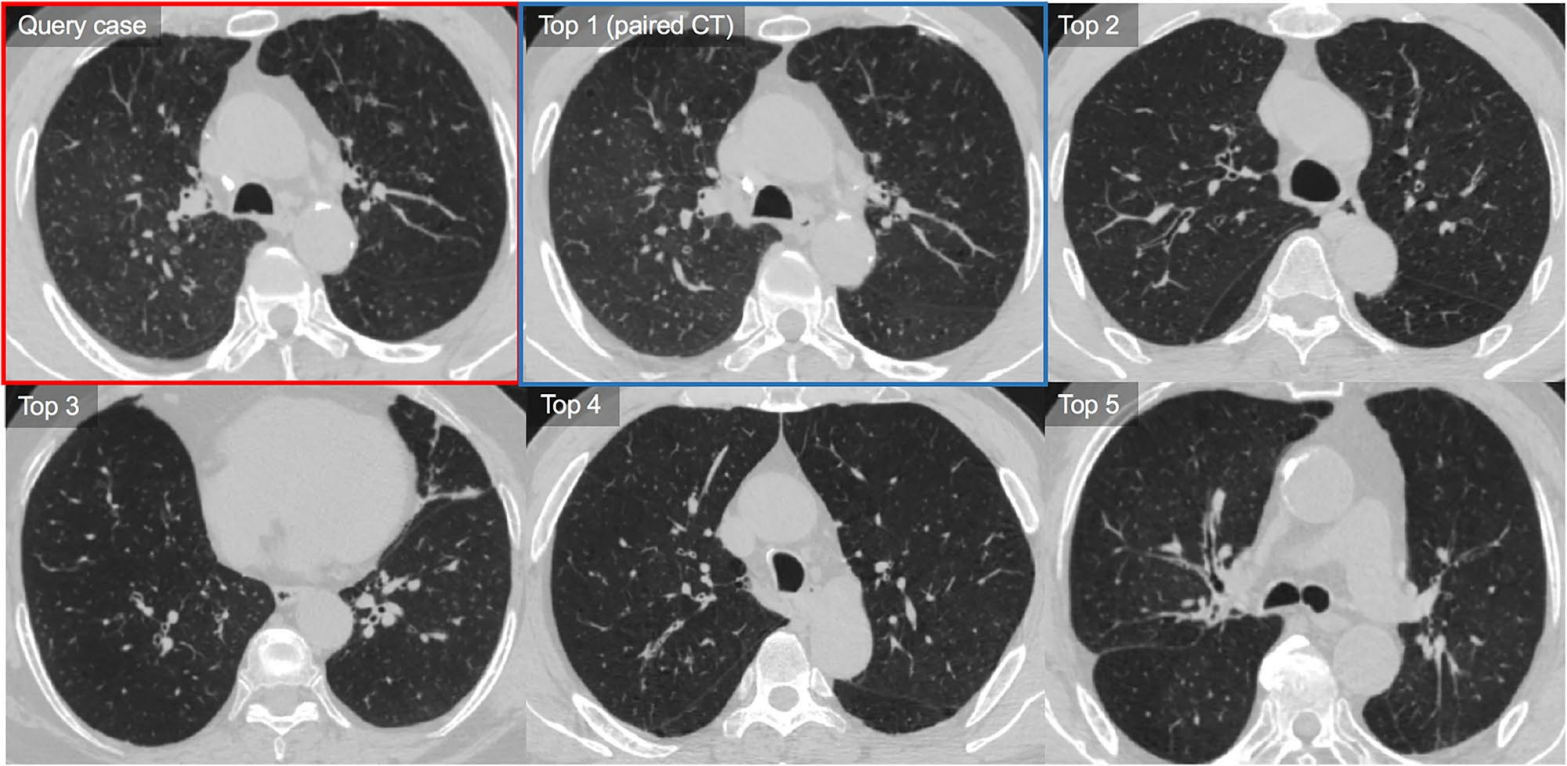
Representative case of the use of the content-based image retrieval (CBIR) system for chronic obstructive pulmonary disease. The query CT shows diffuse bronchial wall thickening in both lungs with multifocal mosaic parenchymal attenuation (Pi10 = 7.1, PVV = 0.55, LAA = 2.9 and D-slope = 3.9). CBIR system retrieves five similar CTs based on the measured similarity in descending order. The query case is outlined with a red box and its paired case (a pair of baseline CT without interval change compared with the query case [follow-up CT]) among the retrievals is outlined with a blue box. Our CBIR system retrieved the baseline pair of query CTs from the database as the top 1 retrieval. CT images of top 2 to top 5 retrievals also show quite similar CT features showing bronchial wall thickening with or without endobronchial mucus plugging and mosaic parenchymal attenuation. No retrieval CT cases demonstrates more than moderate extent of emphysema. In the visual similarity assessment, two chest radiologists rated the similarity scores of the top 1 to 5 as 5, 5, 4, 4, and 4, respectively, and the other chest radiologist as 5, 4, 5, 4, and 4.

Among the retrieved cases, 64.8% (53.2% for reader 1 and 76.4% for reader 2) showed similarity scores of 4 or 5 as rated by two radiologists (Table 4 and Fig. 5). At least one case scored 5 points in 74% (74/100; 76% [38/50] for reader 1 and 72% [36/50] for reader 2) of all 50 query cases, and at least one case scored four or more points in 98% (49/50) and 100% (50/50) of all 50 query cases rated by two radiologists, respectively. The mean similarity scores of the top 1–5 retrieved CT scans rated by the two radiologists were 3.8 ± 0.05 (3.62 ± 0.06 for reader 1 and 3.99 ± 0.05 for reader 2, respectively. The interreader agreement for the similarity scores were moderate (weighted κ = 0.52, 95% confidence interval [CI] = 0.43–0.61; ICC = 0.68, 95%CI = 0.51–0.79).

**Table 4:** Similarity scores of the top 1–5 retrieved computed tomography (CT) images.

Number of retrieval order	CT scans with similarity scores ≥ 4			Mean similarity score*		
	Reader 1	Reader 2	Overall	Reader 1	Reader 2	Overall
Top 1	74.0 (37/50)	88.0 (44/50)	81.0	4.16 ± 0.15	4.34 ± 0.10	4.25 ± 0.11
	(CI 60.2–84.3)	(CI 75.8–94.5)	(CI 70.8–88.3)	(CI 3.87–4.45)	(CI 4.14–4.54)	(CI 4.04–4.46)
Top 2	64.0 (32/50)	82.0 (41/50)	73.0	3.74 ± 0.11	4.08 ± 0.10	3.91 ± 0.09
	(CI 49.9–76.0)	(CI 68.9–90.4)	(CI 61.9–81.8)	(CI 3.53–3.95)	(CI 3.89–4.27)	(CI 3.74–4.08)
Top 3	48.0 (24/50)	82.0 (41/50)	65.0	3.60 ± 0.14	4.02 ± 0.09	3.81 ± 0.10
	(CI 34.6–61.7)	(CI 68.9–90.4)	(CI 54.0–74.6)	(CI 3.33–3.87)	(CI 3.84–4.20)	(CI 3.61–4.01)
Top 4	46.0 (23/50)	64.0 (32/50)	55.0	3.38 ± 0.13	3.80 ± 0.10	3.59 ± 0.10
	(CI 32.8–59.8)	(CI 49.9–76.0)	(CI 43.1–66.4)	(CI 3.12–3.64)	(CI 3.60–4.0)	(CI 3.40–3.78)
Top 5	34.0 (17/50)	66.0 (33/50)	50.0	3.24 ± 0.12	3.72 ± 0.09	3.48 ± 0.09
	(CI 22.3–48.0)	(CI 52.0–77.7)	(CI 39.1–60.9)	(CI 3.01–3.47)	(CI 3.54–3.90)	(CI 3.31–3.65)
Overall	53.2	76.4	64.8	3.62 ± 0.06	3.99 ± 0.05	3.81 ± 0.05
	(CI 46.1–60.2)	(CI 69.5–82.1)	(CI 58.8–70.4)	(CI 3.50–3.75)	(CI 3.90–4.09)	(CI 3.71–3.91)

Unless otherwise indicated, data are percentage, with numerator and denominator in parentheses.

*Data are mean ± standard error. 95% CIs were calculated using GEE with logit and identified link function.

CI, 95% confidence interval.

## Discussion

This study developed a CBIR system for patients with COPD that incorporated quantitative CT features of the lungs, airway, and pulmonary vessels. This CBIR system showed good performance and retrieved the same pairs of query CT scans as the top 1–5 retrievals in 34 of 50 queries (68.0%). Among the retrieved cases, 64.8% (324/500) showed visual similarity scores of 4 or 5 and at least one case scored 5 points in 74% (74/100) in all query cases. The mean similarity scores of the top 1–5 retrieved CT scans rated by the two radiologists were 3.8 ± 0.05.

Few studies have used CBIR as a diagnostic tool to interpret chest CT scans. Moreover, no dedicated CBIR system has yet been described for the evaluation of obstructive lung diseases^19,26,27^. Oosawa et al. and Aisen et al. respectively, developed CBIR systems for various respiratory diseases including emphysema and other disease categories such as infectious diseases and pneumothorax^26,27^. Although both studies included emphysema, the CBIR systems were not developed for the evaluation of obstructive lung diseases but rather to aid in the differential diagnosis of various respiratory diseases on chest CT. Moreover, the numbers of each disease in the databases and test cases were too small to evaluate the performance of CBIR. In addition, the criteria for assessing the visual similarities between query and retrieved CT scans were subjective and not precisely evaluated. A major challenge of CBIR as a diagnostic tool is identifying relevant and effective features and distance measures that match clinician requirements based on a particular application. Thus, CBIR research has aimed to reduce the semantic gap between image feature representation and human visual understanding to achieve better retrieval accuracy in terms of clinical relevance. In our study, by applying automated quantitative CT measures including emphysema, bronchial wall thickening and pulmonary vessels as features for CBIR, patients in obstructive lung diseases with similar phenotypes can be objectively identified in the database and easily visualized on our CBIR system.

The similar image retrieval can support clinical decision-making by offering patients with known clinical outcomes, including treatment response or prognosis with similar imaging characteristics, a second opinion in the management of patients with obstructive lung diseases. During the past decade, the Global Initiative for Obstructive Lung Disease (GOLD) therapeutic strategy acknowledged the limitations of using spirometry alone to guide therapy by assessing the disease severity^28^. To address the complexity of COPD, identification of clinical phenotypes as clusters of patients with similar clinical characteristics, prognosis and/or therapeutic needs has emerged as an important approach to guide personalised medicine. However, many COPD subtypes have been proposed, but there is still no consensus about how many subtypes there are and how they should be objectively and reproducibly classified^3^. CBIR can be another approach to precision medicine for individualized therapy. Evaluating similar cases offers a slightly different perspective than directly knowing which cluster a case belongs based on cluster analysis (i.e., k-means clustering). Specifically, it can provide more granular details that are especially pertinent to patients who lie on the boundaries of distinct clusters. If the database is expansive and the features underpinning the similarity between cases are meticulously curated, CBIR can retrieve and showcase individual cases that mirror even closer characteristics within the same cluster group, surpassing the depth provided by standalone cluster analysis. CBIR stands out, especially when compared with other decision-support tools, for its intensive human-AI interaction. Depending on the interface, retrieval accuracy, and the presented linked information, CBIR can foster increased trust and utility for AI-based decision supporting tools, making it an invaluable human-centered tool^29^. When a clinician is deciding follow-up strategies or determining which pharmacological treatment options to prescribe to a certain patient, by applying CBIR, the treatment effect and clinical outcomes of similar patients retrieved from the CBIR system can be demonstrated and easily reviewed to facilitate decision making whether the disease is reversible, to assess whether the disease will be refractory to standard therapy or benefit from biologics, and to estimate the risk of recurrent episodes of exacerbation. Incorporating clinical variables such as age, sex, smoking, lung function and comorbidities for calculating similarity index may expand the potential of CBIR and able to bring more meaningful retrievals of similar cases strongly associated with clinical outcomes. Furthermore, those similarity indices might help to discover new important phenotype in patients with COPD.

To build the CBIR system for obstructive lung diseases, we selected four quantitative features including emphysema, bronchial wall thickening, and peripheral pulmonary vasculature, to estimate the similarity distances between cases. Quantitative CT evaluation has been validated as a tool for the assessment of the presence and severity of emphysema, expiratory airflow obstruction, and airway wall thickening^30–33^. These measures included EI or LAA_−950_ for emphysema extent and severity and Pi10 for airway wall thickness, which were well validated in multiple studies and showed strong associations with spirometric results and survival^34–36^. The D-slope, which is a measure representing the distribution of emphysema hole-size, was significantly correlated with clinical parameters such as FEV1, diffusion capacity, exercise capacity, and quality of life^37^. Regarding pulmonary vessels, pulmonary vascular remodeling in smokers is characterized by distal pruning of the blood vessels, which can be automatically identified, segmented, and quantified, including measures such as total blood vessel volume or numbers and the aggregate vessel volume for vessels < 5 mm^2^^25,38^. The present study used VN_<5 mm_ (number of vessels with area < 5 mm^2^ in the theoretical lung surface area at a depth of 12 mm from the pleural surface) as a measure of pulmonary vascular alteration, which was significantly correlated with FEV1 and FEV1 to FVC ratio and extent of emphysema^25^. Our CBIR system showed good performance, achieving a 68.0% (34 of 50) rate of retrieving the same paired CT images from the same patients. While the selected features used in our CBIR system have been verified in various studies and also showed good performance in our study, further investigations are needed regarding methods to identify features that accurately reflect the clinical course and retrieve clinically meaningful similar cases.

Though our CBIR system successfully retrieved the same paired CT with a query CT, it would be relevant to confirm whether radiologists will interpret the retrieved CT as similar images. The mean similarity scores of the top 1–5 retrieved CT scans rated by two radiologists were 3.62 and 3.99, respectively, which is close to 4, indicating that more than two or three features were similar. For the top-one and top-two retrievals, the visual similarity score was 4 or more than four in 82% and 73% of total query cases, respectively. Moreover, for the overall scores of the two readers, the similarity scores were also consistent with the rank of retrievals based on the similarities given by the CBIR system. As visual similarity is subjective, assessment of the interobserver variability of the visual similarity scores showed moderate agreement between the readers. Therefore, the CBIR system produced reasonable results for retrieving similar cases in patients with obstructive lung diseases that radiologists could also agree on.

Our study has several limitations. First, we did not incorporate all quantitative CT features; rather, we included five well-established features for whole lung volume. As the recent research for CBIR is also shifting to the use of deep neural networks, incorporating large-scale quantitative feature datasets and clinical features by applying unsupervised learning methods might further improve the CBIR performance^18^. Second, as the data were not available, we could not link the patients’ characteristics to clinical outcomes such as treatment response or survival. Third, our dataset is derived from the Korean Obstructive Lung Disease (KOLD) cohort which ran from May 2005 to October 2013. This period predates the standardized CT protocols proposed by SPIROMICS. During this time, the tube voltage for the standardized protocol of the registry was set to 140 kV, while SPIROMICS recommended 120 kV^39^. Using 140 kV might not be optimal; however, the single threshold of -950 HU remains a widely accepted measure for emphysema quantification and has been prevalently employed in prior research^40,41^. In addition, previous studies also demonstrated that different tube voltage, such as 120 kV and 140 kV, had minimal impact on the CT number, air densities and/or CT numbers less than 0 HU compared with effect of vendors^42,43^. Therefore, analyzing emphysema at 140 kV with -950HU is less likely to have significant effect. There can be the potential variability introduced by different CT protocols, which could indeed impact the sensitivity of emphysema quantification. However, given that all scans in the KOLD cohort adhered to a consistent 140 kV using standardized protocols, the variability in measurements might be minimal, ensuring a consistent assessment of feature similarity across the patients in the datasets. Finally, as this was a pilot study, we did not evaluate the clinical impact of CBIR for the diagnosis of obstructive lung diseases, including whether it can aid in the diagnosis of COPD phenotype and improve the clinical consequences by changing the diagnosis. In fact, evaluating the performance of the CBIR is challenging due to a lack of sufficient validated evaluation matrices. Consequently, clinical validation becomes paramount, assessing its impact on diagnosis and treatment. Therefore, what is needed for the future use of this method a multicenter collaborative approach to build a cloud-based lung image library or quantitative imaging database for COPD with clinical information and outcomes (treatment effect, exacerbation, and death) that is easily accessible and evaluating clinical impact of CBIR in diagnosis and management of obstructive pulmonary disease. Such a collective effort would not only enhance efficiency and accuracy of CBIR but also make it more applicable in clinical settings. Furthermore, promoting data availability and fostering collaborations would inevitably propel the research in this domain.

In conclusion, the proposed CBIR system for obstructive lung diseases integrating quantitative CT measures demonstrated potential for retrieving chest CT scans with similar phenotypic imaging characteristics. Applying CBIR in the obstructive lung diseases with further linking of clinical outcomes of similar cases may aid in the assessment of those patients to establish a treatment plan and predict prognosis.

### Supplementary Information

Supplementary Information.

## Data Availability

The datasets generated or analyzed during the study are available from the corresponding author on reasonable request.
