# Supplementary material for: Evaluation of retrieval accuracy and visual similarity in content-based image retrieval of chest CT for obstructive lung disease
Source: Sci Rep. 2024 Feb 26;14:4587. doi: 10.1038/s41598-024-54954-5 (PMC10894863; doi:10.1038/s41598-024-54954-5)
Supplement: Supplementary file 1 — Supplementary Information. [file 41598_2024_54954_MOESM1_ESM.docx]

**Supplementary material**

**Study cohorts – KOLD cohort**

All subjects were selected from the Korean Obstructive Lung Disease (KOLD) Cohort Study, which were prospective longitudinal studies of patients with obstructive lung disease from the pulmonary clinics of 17 centers in South Korea. The KOLD cohort study included patients from May 2005 to October 2013. The details of the KOLD cohort study have been published previously.^24^ Briefly, the study cohort enrolled patients aged >18 years with chronic respiratory symptoms as well as one or both of airflow limitation or bronchial hyper-responsiveness. Airflow limitation was defined as a prebronchodilator forced expiratory volume in 1 second (FEV1) over forced vital capacity (FVC) value of <0.7. Bronchial hyper-responsiveness was measured using the methacholine provocation test and was defined as the provocative concentration that reduced the FEV1 by 20% <16 mg/mL. To allow the inclusion of asthma and other unclassified obstructive lung disease patients in the study, the KOLD Cohort Study did not use smoking history as an inclusion criterion. Moreover, patients with bronchial hyper-responsiveness were included and a prebronchodilator FEV1/FVC<0.7 was used rather than postbronchodilator FEV1/FVC<0.7. The exclusion criteria were presence of co-existing diseases that would interfere with the study results, including malignancy and respiratory disease other than obstructive disease. Patients with a recent exacerbation or other respiratory diseases such as pneumonia were also excluded. The present study was approved by the Asan Medical Center Institutional Review Board (No. 2017-1067) and by the Institutional Review Boards of the other 16 participating hospitals. Written informed consent was obtained from all patients.

**Testing the air trapping index (ATI) for CBIR**

To assess small airway disease, the air trapping index (ATI) also can be analyzed by comparing the densities using co-registration of inspiration and expiration CT scans. In the ATI, air trapping is defined as an area with a density increase of <60 HU between inspiration and expiration CT scans (1, 2).

In our initial tests, when we incorporated the ATI for the whole lung volume alongside our final set of four feature vectors, we observed a decline in the retrieval rates for exact pairs of query CTs. Specifically, the rates were 24.0% (12/50) for the top-three retrievals and 30.0% (15/50) for top-five retrievals, respectively.

Therefore, since expiratory CTs are not customarily obtained in daily clinical practice, this could also substantially narrow the applicability of the system. Considering the aforementioned reasons and the fact that our KOLD cohort-based CBIR database had a limited number of patients with asthma, we decided against the inclusion of the ATI in our final CBIR model.

Reference

1. Kim EY, Seo JB, Lee HJ, Kim N, Lee E, Lee SM, et al. Detailed analysis of the density change on chest CT of COPD using non-rigid registration of nspiration/expiration CT scans. Eur Radiol 2015;25:541-549.
2. Lee SM, Seo JB, Lee SM, Kim N, Oh SY, Oh YM. Optimal threshold of subtraction method for quantification of air-trapping on coregistered CT in COPD patients. Eur Radiol 2016;26:2184-2192.
